# A Novel RNA-Binding Protein Signature to Predict Clinical Outcomes and Guide Clinical Therapy in Gastric Cancer

**DOI:** 10.3389/fmed.2021.670141

**Published:** 2021-07-15

**Authors:** Zhigang Qiu, Haitao Jiang, Kun Ju, Xichun Liu

**Affiliations:** ^1^Department of Gastrointestinal Surgery, The Affiliated Hospital of Qingdao University, Qingdao, China; ^2^Department of Oncology, Qingdao Municipal Hospital, Qingdao, China; ^3^Department of Emergency, The Affiliated Hospital of Qingdao University, Qingdao, China

**Keywords:** gastric cancer, RNA binding protein, signature, immune microenvironment, chemosensitivity, nomogram

## Abstract

**Objective:** This study aimed to develop an RNA-binding protein (RBP)-based signature for risk stratification and guiding clinical therapy in gastric cancer.

**Methods:** Based on survival-related RBPs, an RBP-based signature was established by LASSO regression analysis in TCGA dataset. Kaplan–Meier curves were drawn between high- and low-risk groups. The predictive efficacy of this signature was assessed via ROCs at 1-, 3-, and 5-year survival. Its generalizability was verified in an external dataset. Following adjustment with other clinicopathological characteristics, the independency of survival prediction was evaluated via multivariate Cox regression and subgroup analyses. GSEA was utilized in identifying activated pathways in two groups. Stromal score, immune score, tumor purity, and infiltration levels of 22 immune cells were determined in each sample via the ESTIMATE and CIBERSORT algorithms. The sensitivity to chemotherapy drugs was assessed through the GDSC database.

**Results:** Data showed that patients with high risk exhibited unfavorable clinical outcomes than those with low risk. This signature possessed good performance in predicting 1-, 3-, and 5-year survival and can be independently predictive of patients' survival. Calcium, ECM receptor interaction, and focal adhesion were highly enriched in high-risk samples. High-risk samples presented increased stromal and immune scores and reduced tumor purity. Moreover, this signature presented close relationships with immune infiltrations. Low-risk specimens were more sensitive to sorafenib, gefitinib, vinorelbine, and gemcitabine than high-risk specimens.

**Conclusion:** This RBP-based signature may be a promising tool for predicting clinical outcomes and guiding clinical therapy in gastric cancer.

## Introduction

Gastric cancer ranks fifth in incidence and third mortality among global cancers (1, 2). Patients are diagnosed in histology following endoscopic biopsy and staged by computed tomography, endoscopic ultrasound, positron emission tomography, or laparoscopy (3). This cancer is a highly heterogeneous disease at the molecular and phenotypic levels. Subjects diagnosed by the same TNM stage and treated by similar therapeutic regimens present varied prognoses, emphasizing that TNM stage by itself cannot provide complete prognostic information (4). Endoscopic surgery is a primary therapeutic method for early subjects. Nevertheless, most patients are diagnosed at an advanced stage, who have missed the optimal time for surgery. Despite adjuvant chemotherapy, immunotherapy, and targeted therapy, advanced subjects' median survival time is <1 year (5). Hence, innovative strategies are required for boosting risk stratification as well as predictive accuracy of clinical outcomes.

RNA-binding proteins (RBPs), a type of protein, may be interacted with a variety of RNAs. At present, 1,542 human RBP genes have been found, which participate in posttranscriptional modulation such as RNA splicing, polyadenylation, editing, modification, and translation (6–8). Aberrant expression of RBPs may induce progress of various malignancies, including gastric cancer (9, 10). RBPs have been detected to widely express in tumor cells, thereby affecting the translation of mRNAs into proteins and carcinogenesis processes (11). Increasing evidence has highlighted clinicopathologic implication of immune microenvironment in survival outcomes and therapeutic efficacy in gastric cancer (12). Recent findings have found that RBPs may affect immune microenvironment across different cancer types (13). For example, RBP SORBS2 inhibits metastatic colonization of ovarian cancer through enhancing stability of tumor-suppressive immunomodulatory transcripts (13). In-depth understanding of the roles of RBPs will offer innovative ideas for immunotherapy of gastric cancer. Previously, Huang et al. (14) proposed a 6-RBP signature that predicted the survival of hepatocellular carcinoma with high accuracy. Li et al. (15) developed a 9-RBP signature with accurate predictive efficacy for lung squamous cell carcinoma patients' prognosis. However, there is still lack of gene signature based on RBPs for gastric cancer. Furthermore, the relationships between RBPs and immune microenvironment are required for further analysis. Here, this work developed and verified an RBP-based model that exhibited a good performance in predicting patients' survival and was significantly associated with immune microenvironment using public datasets.

## Materials and Methods

### Gastric Cancer Datasets Acquiring and Preprocessing

Transcriptome FPKM RNA-seq profiles of gastric cancer were retrieved from the Cancer Genome Atlas (TCGA; https://tcga-data.nci.nih.gov/tcga/). Meanwhile, clinical information, including age, gender, grade, stage, TNM, and overall survival, was acquired by the UCSC Xena (https://xena.ucsc.edu/). The details are listed in Supplementary Table 1. After excluding samples with survival time of 0, 350 cases of gastric cancer specimens were retained as a training set. FPKM values were converted to TPM values for normalization (16). In the Gene Expression Omnibus (GEO; https://www.ncbi.nlm.nih.gov/gds/) repository, the GSE84437 dataset was obtained on the GPL6947 platform (17). A total of 431 samples with survival time >0 were utilized as a validation set. The clinical information is shown in Supplementary Table 2. Based on a previous published study, 1,542 RBPs were retrieved (Supplementary Table 3) (18).

### Establishment and Validation of a Prognostic RNA-Binding Protein Gene Signature

Univariate Cox regression analyses were employed for analyzing associations between RBPs and clinical outcomes of gastric cancer. Prognosis-related RBPs with *p* < 0.05 were retained. Then, least absolute shrinkage and selection operator (LASSO) regression analyses were adopted to acquire key prognostic RBPs (19). The risk scores of subjects were determined following the formula: risk score = Σ expression level of gene_i_^*^ β_i_. β represents the regression coefficient of gene_i_. Then, the median value was utilized as the cutoff value. Subjects were separated into high- and low-risk subgroups. Utilizing Kaplan–Meier curves, survival probability between the two groups was compared by log-rank test. Receiver operating characteristic curves (ROCs) for 1-, 3-, and 5-year survival were conducted via the ROC package in R. Area under the curve (AUC) was then determined. With the same cutoff value, predictive efficacy of the RBP gene signature was validated in the verification set.

### Protein–Protein Interaction Analysis

Functional associations between prognosis-related RBPs were predicted by the STRING online database (http://www.bork.embl-heidelberg.de/STRING/) (20).

### Univariate and Multivariate Cox Regression Analyses

To analyze the relationships between clinical factors (age, gender, grade, stage, TNM, and risk score) and survival, univariate Cox regression analyses were carried out in the training and verification sets, separately. The independency of survival prediction of clinical factors was evaluated via multivariate Cox regression analyses. Hazard ratio (HR), 95% confidence interval (CI), and *p* values were calculated, respectively.

### Subgroup Analyses

Patients were separated into different subgroups on the basis of different clinicopathological characteristics, including age (>65 and ≤ 65), gender (female and male), grade (grades 1–2 and grade 3), stage (stages I–II and stages III–IV), T (T1–2 and T3–4), N (N0 and N1–3), and M (M0 and M1). Kaplan–Meier curves followed by log-rank test were presented between high- and low-risk subjects in above subgroups.

### Pathway Enrichment Analysis

The gene set enrichment analysis (GSEA) 4.0.3 software was utilized in identifying activated signaling pathways in high- and low-risk subgroups (21, 22). This study retrieved the hallmark gene set (h.all.v6.0.symbol.gmt) from the Molecular Signatures Database as a reference gene set. Enriched pathways were screened according to nominal *p* < 0.05 and adjusted *p* < 0.05.

### Estimation of Stromal Score, Immune Score, and Tumor Purity

Stromal score, immune score, and tumor purity for each specimen were evaluated via the Estimation of STromal and Immune cells in MAlignant Tumour tissues using Expression data (ESTIMATE) algorithm (23). The differences in stromal score, immune score, and tumor purity between the two subgroups were compared through the Wilcoxon rank-sum test. Kaplan–Meier curves were conducted for estimating survival differences between different subgroups, as follows: high vs. low stromal score, high vs. low immune score, and high and low tumor purity.

### Assessment of Immune Cell Infiltration

The infiltration levels of 22 immune cell types were quantified in gastric cancer specimens utilizing the Cell type Identification by Estimating Relative Subsets of RNA Transcripts (CIBERSORT) algorithm as well as the LM22 gene sets containing 547 markers (24). The comparison of immune cell types between the high- and low-risk groups was carried out through the Wilcoxon rank-sum test.

### Estimation of Immune Checkpoint Expression

The expression levels of 47 immune checkpoints were estimated in gastric cancer samples. Their expression was compared in the high- and low-risk groups by the Wilcoxon rank-sum test.

### Drug Sensitivity Assessment

The sensitivity to different chemotherapy drugs for each sample was estimated through the Genomics of Drug Sensitivity in Cancer (GDSC; https://www.cancerrxgene.org/) database (25). The calculation of half maximal inhibitory concentration (IC_50_) was achieved through the pRRophetic package in R (26).

### Construction a Prognostic Nomogram Model

A nomogram model construction was achieved by the rms package as well as the survival package in R. This nomogram contained independent prognostic factors. Calibration curves were then depicted for evaluation of the predictive potency for 1-, 3-, and 5-year clinical outcomes of this nomogram.

### Statistical Analyses

All analyses were achieved by available packages in R language 3.4.1 (http://www.R-project.org). Comparisons between the two groups were performed by the Wilcoxon rank-sum test or Student' *t*-test. Values of *p* < 0.05 indicated statistical significance.

## Results

### Construction of a Prognostic Signature for Gastric Cancer

**Figure 1:**
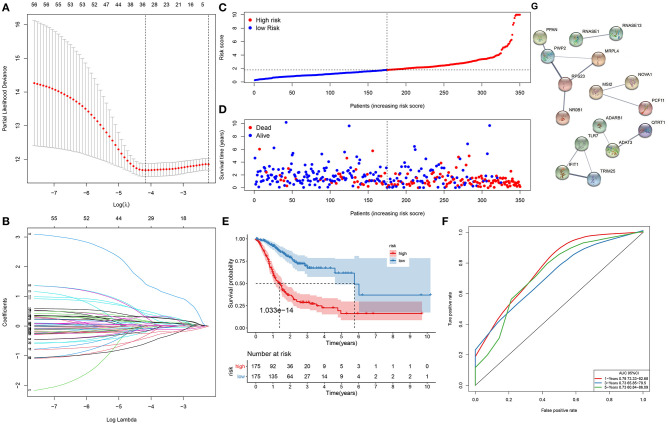
A gene signature for predicting the survival of gastric cancer patients. **(A)** The optimal parameter (λ) is where the vertical dotted line is. **(B)** Regression coefficients for RNA-binding proteins (RBPs) when determining the optimal parameter (λ). Each curve represents the change in trajectory of coefficients for each independent variable, and the ordinate indicates the coefficient values. **(C)** The distribution of risk scores for all subjects. The vertical dotted line corresponds to the median value of risk scores. **(D)** Survival status for all subjects ranked by risk scores. **(E)** Kaplan–Meier curves of overall survival between high- and low-risk subjects. **(F)** Receiver operating characteristic curves (ROCs) of 1-, 3-, and 5-year survival. **(G)** A protein–protein interaction (PPI) network based on the 33 key RBP genes.

Herein, 350 gastric cancer specimens were employed as the training set. Totally, 58 RBPs exhibited significant associations with survival of gastric cancer patients (Table 1). To avoid data overfitting, coexpressed RBPs were eliminated through LASSO regression analyses (Figures 1A,B). Consequently, 33 key RBPs were retained for establishment of a prognostic signature. We determined the risk scores of all subjects. Table 2 listed the regression coefficients of these key RBPs. Then, these subjects were separated into high- and low-risk groups (Figure 1C). In Figure 1D, the number of dead patients in the high-risk group was significantly higher than that in the low-risk group. The difference in survival between groups was compared in depth. Figure 1E displayed that subjects with high risk often experienced more unfavorable survival time than those with low risk (*p* = 1.033e−14). Following confirmation by ROCs, the AUCs for 1-, 3-, and 5-year clinical outcomes were separately 0.779, 0.759, and 0.788 (Figure 1F). These data were indicative of the predictive potential of the signature. To observe the interactions between 33 key RBPs, we constructed a PPI network. In Figure 1G, 14 key RBPs had mutual regulation.

**Table 1:** Univariate Cox regression analyses of survival-related RBPs in gastric cancer.

**RBPs**	**HR**	**HR.95L**	**HR.95H**	***P***	**RBPs**	**HR**	**HR.95L**	**HR.95H**	***P***
DAZAP1	0.6867	0.4886	0.9651	0.0304	TRIM25	0.6839	0.5358	0.873	0.0023
MSI2	0.7234	0.5639	0.9279	0.0108	ZFP36	1.2598	1.0622	1.4943	0.008
RBMS1	1.3013	1.0761	1.5735	0.0066	PURG	2.2696	1.11	4.6405	0.0247
RBMS3	1.248	1.0654	1.4619	0.0061	TSEN54	0.7761	0.6073	0.9919	0.0429
METTL2B	0.6348	0.4591	0.8777	0.006	EZH2	0.8015	0.6591	0.9746	0.0266
AKAP8	0.6722	0.4699	0.9618	0.0298	QKI	1.3028	1.0735	1.5811	0.0074
REPIN1	0.7522	0.6048	0.9356	0.0105	ISY1	0.658	0.4371	0.9905	0.0449
DZIP1	1.2199	1.0257	1.4509	0.0246	PPAN	0.735	0.5451	0.9911	0.0436
DYNLL1	1.5709	1.0392	2.3746	0.0322	RAVER1	0.7913	0.6337	0.9881	0.0389
GLE1	0.7245	0.5553	0.9452	0.0175	ENOX1	1.2325	1.0315	1.4726	0.0214
BOLL	1.8406	1.1722	2.8899	0.008	RNASE11	24.976	1.6554	376.83	0.0201
FBXO17	1.1401	1.0076	1.2899	0.0374	TRMT1	0.7019	0.5141	0.9584	0.0259
PWP2	1.3298	1.0832	1.6325	0.0065	RNASE13	13.306	2.5541	69.315	0.0021
MRPL4	0.7253	0.5586	0.9419	0.016	FTO	1.3563	1.0175	1.808	0.0377
QTRT1	0.7165	0.5246	0.9785	0.036	FAM98C	0.7202	0.5215	0.9947	0.0464
GTPBP3	0.7488	0.5634	0.9952	0.0463	PCF11	0.7254	0.5332	0.9869	0.041
SMAD5	1.3215	1.0084	1.7319	0.0433	TTF2	0.7462	0.5723	0.9731	0.0307
ADARB1	1.2343	1.011	1.5069	0.0387	TLR7	1.2283	1.0284	1.4669	0.0232
POLRMT	0.7285	0.5607	0.9466	0.0178	PABPC5	1.5082	1.0935	2.0801	0.0123
LENG9	0.8052	0.6575	0.9862	0.0362	RBM15	0.7482	0.5637	0.9932	0.0448
RNASE9	8.1851	1.8494	36.225	0.0056	NR0B1	3.9608	1.7366	9.0333	0.0011
REXO1	0.7606	0.5846	0.9895	0.0415	RPS4Y2	1.8092	1.1338	2.8869	0.0129
NXF5	8.1997	1.7738	37.904	0.0071	ADAT3	0.7003	0.5595	0.8764	0.0019
PEG10	1.1012	1.018	1.1912	0.0162	BICC1	1.208	1.0468	1.3941	0.0097
RNASE1	1.2357	1.0853	1.407	0.0014	SURF6	0.7307	0.5512	0.9685	0.0291
RNASE2	1.2156	1.0392	1.4219	0.0147	RPS23	1.3576	1.0036	1.8364	0.0473
RNASE3	1.3758	1.112	1.7022	0.0033	IFIT1	1.1369	1.005	1.2862	0.0414
HEXIM2	0.713	0.5087	0.9995	0.0497	NOVA1	1.2027	1.0229	1.4141	0.0255
LARP6	1.2314	1.0523	1.441	0.0094	EIF1AD	0.6806	0.4697	0.9862	0.042

*RBPs, RNA-binding proteins; HR, hazard ratio; HR.95L, lower 95% confidence interval; HR.95H, higher 95% confidence interval*.

**Table 2:** The coefficients of 33 key RBPs in the gene signature.

**Gene**	**Coef**	**Gene**	**Coef**	**Gene**	**Coef**
MSI2	−0.41056215	RNASE3	0.148036367	PCF11	−0.586573788
METTL2B	−0.105183273	HEXIM2	−0.068963889	TTF2	−0.001441001
DYNLL1	0.181641481	TRIM25	−0.151111951	TLR7	0.013281286
BOLL	0.511182302	ZFP36	0.118338975	PABPC5	−0.00140872
PWP2	0.311152879	PURG	−0.02930587	NR0B1	0.319435254
MRPL4	−0.322619794	TSEN54	0.161142621	RPS4Y2	0.301449471
QTRT1	−0.062291791	ISY1	−0.196715792	ADAT3	−0.255476366
SMAD5	0.420293951	PPAN	0.301063239	BICC1	−0.002450685
ADARB1	0.096350867	RNASE13	1.413824833	RPS23	0.121943685
PEG10	0.025434693	FTO	0.364676671	IFIT1	0.036295595
RNASE1	0.147258292	FAM98C	−0.099287095	NOVA1	−0.173519708

*Coef, coefficients*.

### Verification of the Prognostic Signature in an External Dataset

**Figure 2:**
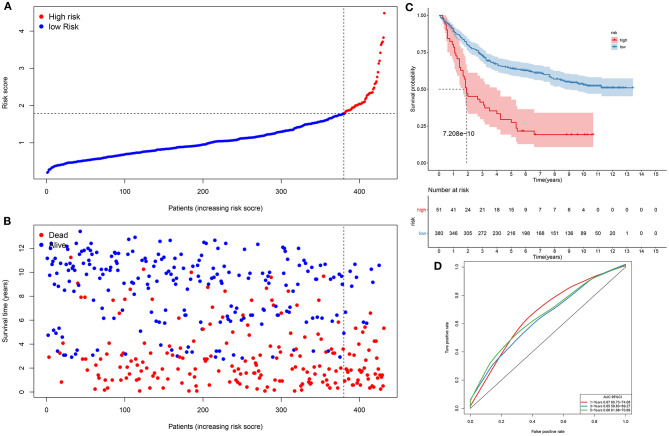
Assessment of this prognostic signature in the verification dataset. **(A)** The ranking of risk scores, and **(B)** the distribution of survival status among gastric cancer samples. The vertical dotted line corresponds to the cutoff of risk scores. **(C)** Kaplan–Meier curves of clinical outcomes concerning high- and low-risk subjects. **(D)** ROCs of 1-, 3-, and 5-year survival.

We further evaluated the generalizability of the signature in the GSE84437 dataset. With the same cutoff value, subjects were separated into high- and low-risk subgroups (Figure 2A). Compared with the low-risk group, there were more patients with dead status in the high-risk group (Figure 2B). Those with high risk presented worse survival time than those with low risk (*p* = 7.208e−10; Figure 2C). The AUCs of 1-, 3-, and 5-year clinical outcomes were separately 0.647, 0.645, and 0.669, which was suggestive that this signature might be used in predicting the patients' survival (Figure 2D).

### The Signature as an Independent Prognostic Factor for Gastric Cancer

**Figure 3:**
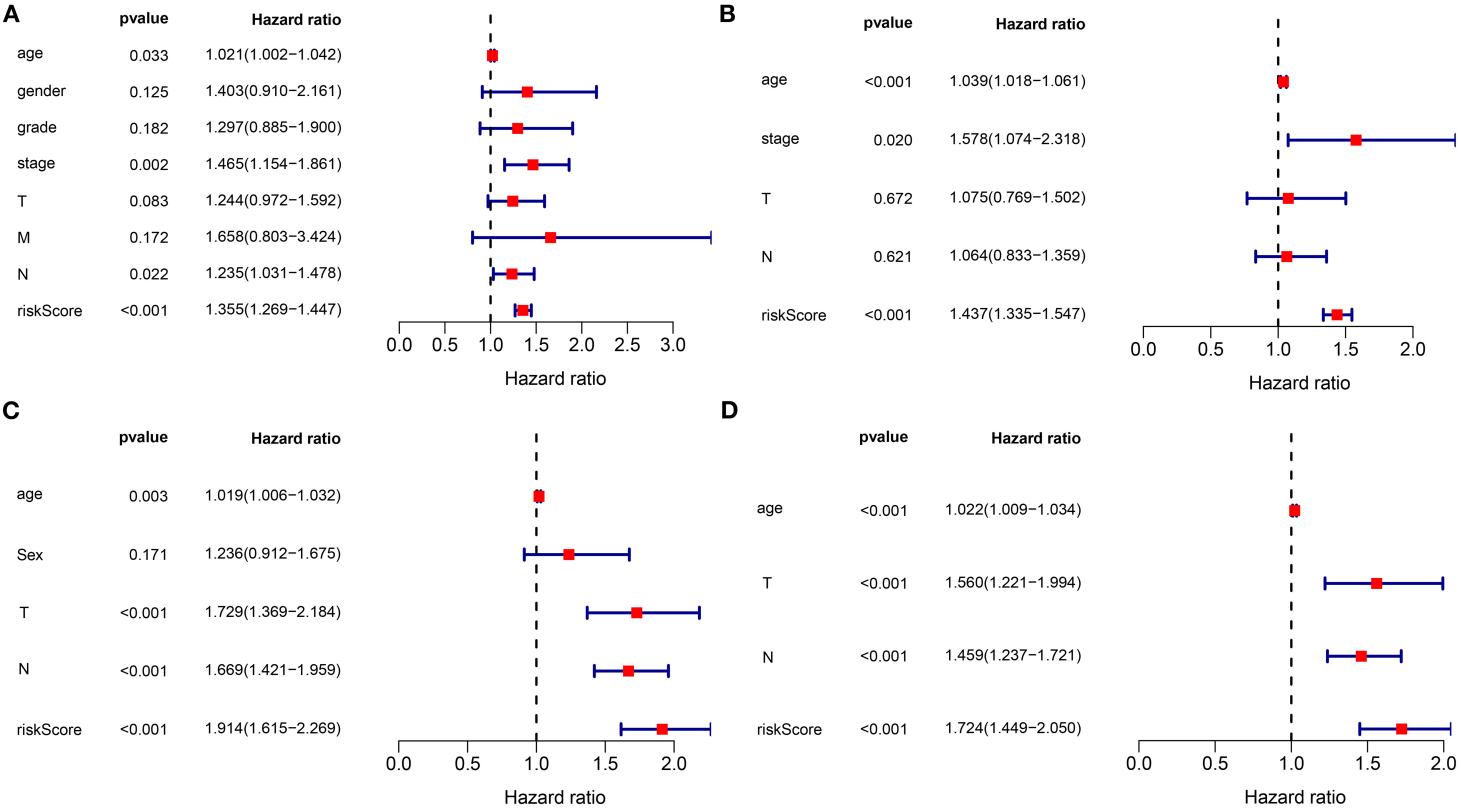
The signature as an independent risk factor of gastric cancer. **(A)** Univariate and **(B)** multivariate Cox regression analyses of age, gender, grade, stage, TNM, as well as signature for gastric cancer in the training set. **(C)** Univariate and **(D)** multivariate Cox regression analyses for age, sex, T, N, and risk score for gastric cancer in the verification set.

In the training set, our univariate Cox regression analyses were indicative that risk score presented a significant correlation with gastric cancer prognosis [*p* < 0.001; HR (95% CI): 1.355 (1.269–1.447)] in Figure 3A. Moreover, age (*p* = 0.033; HR (95%CI): 1.021 (1.002–1.042)], stage (*p* = 0.002; HR (95% CI): 1.465 (1.154–1.861)], and N [*p* = 0.022; HR (95%CI): 1.235 (1.031–1.478)] were also correlated to gastric cancer prognoses. Above were risk factors of gastric cancer. Following multivariate Cox regression analyses, age [*p* < 0.001; HR (95% CI): 1.039 (1.018–1.061)], stage [*p* = 0.020; HR (95% CI): 1.578 (1.074–2.318], and this gene signature [*p* < 0.001; HR (95% CI): 1.437 (1.335–1.547)] independently predicted the patients' survival (Figure 3B). We further verified the independency of the signature in predicting prognosis in an external dataset. Data showed that age [*p* = 0.003; HR (95% CI): 1.019 (1.006–1.032)], T [*p* < 0.001; HR (95% CI): 1.729 (1.369–2.184)], N (*p* < 0.001; HR (95% CI): 1.669 (1.421–1.959)], and this gene signature [*p* < 0.001; HR (95% CI): 1.914 (1.615–2.269)] were risk factors of gastric cancer (Figure 3C). By confirmation of multivariate Cox regression analyses, age [*p* < 0.001; HR (95% CI): 1.022 (1.009–1.034)], T [*p* < 0.001; HR (95% CI): 1.560 (1.221–1.994)], N [*p* < 0.001; HR (95% CI): 1.459 (1.237–1.721)], and this gene signature [*p* < 0.001; HR (95% CI): 1.724 (1.449–2.050)] were independently predictive of clinical outcomes (Figure 3D). Collectively, this signature was an independent risk factor of gastric cancer.

### Subgroup Analysis of the Signature in Predicting Gastric Cancer Patients' Survival

**Figure 4:**
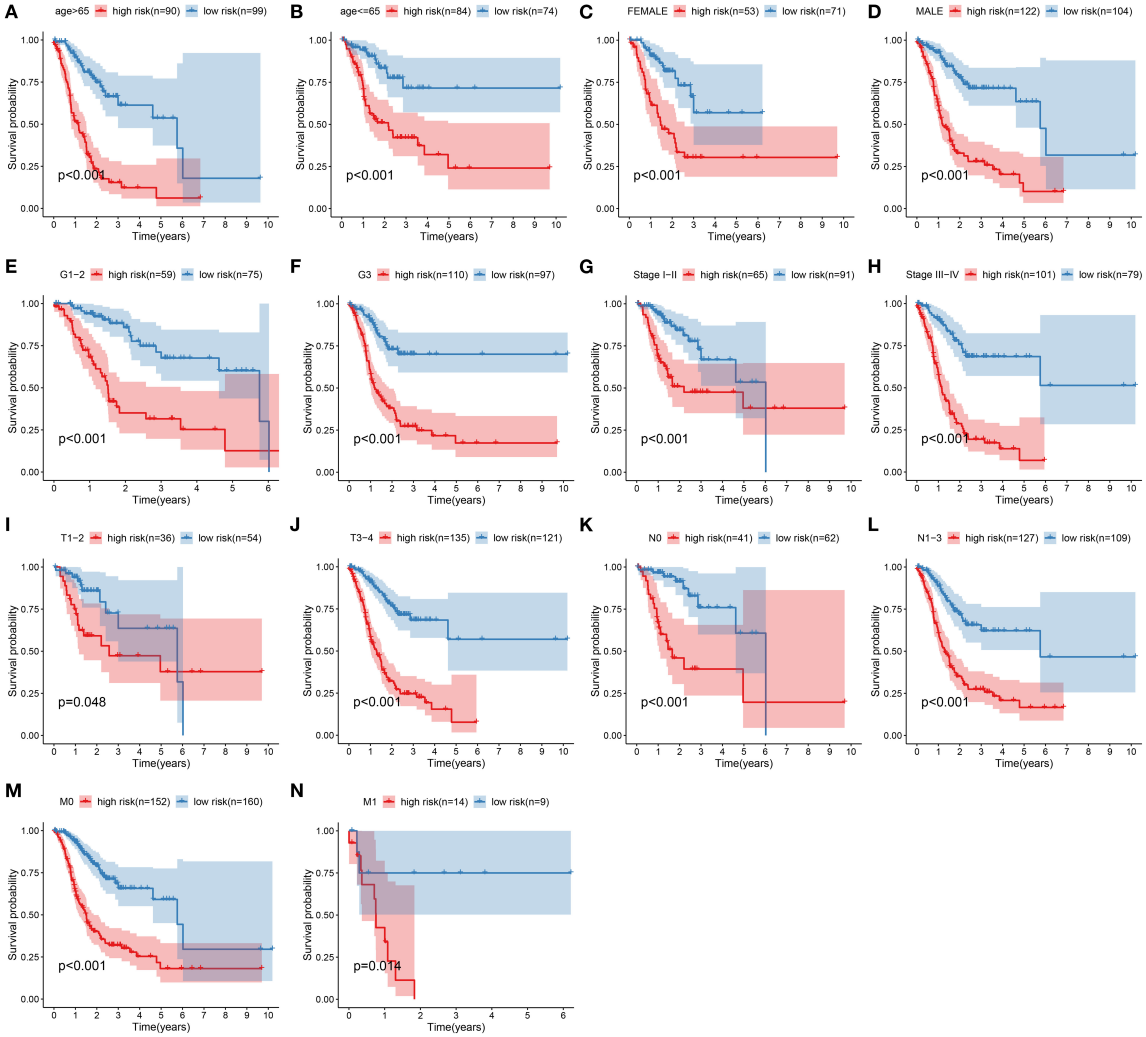
Subgroup analysis of the signature in predicting gastric cancer patients' survival. Kaplan–Meier curves for high- and low-risk subjects in different subgroups including **(A)** age >65, **(B)** age ≤ 65; **(C)** female, **(D)** male; **(E)** grades 1–2, **(F)** grade 3; **(G)** stages I–II, **(H)** stages III–IV; **(I)** T1–2, **(J)** T3–4; **(K)** N0, **(L)** N1–3; **(M)** M0, **(N)** M1.

Subgroup analysis was presented to assess whether the signature was accurately predictive of patients' clinical outcomes in the training set. Data indicated that subjects with high risk were indicative of more unfavorable survival in comparison with those with low risk in different subgroups according to age (>65 and ≤ 65; Figures 4A,B), gender (female and male; Figures 4C,D), grade (G1–2 and G3; Figures 4E,F), stage (stages I–II and III–IV; Figures 4G,H), T (T1–2 and T3–4; Figures 4I,J), N (N0 and N1–3; Figures 4K,L), as well as M (M0 and M1; Figures 4M,N).

### Signaling Pathways Involved in High- and Low-Risk Subgroups

**Figure 5:**
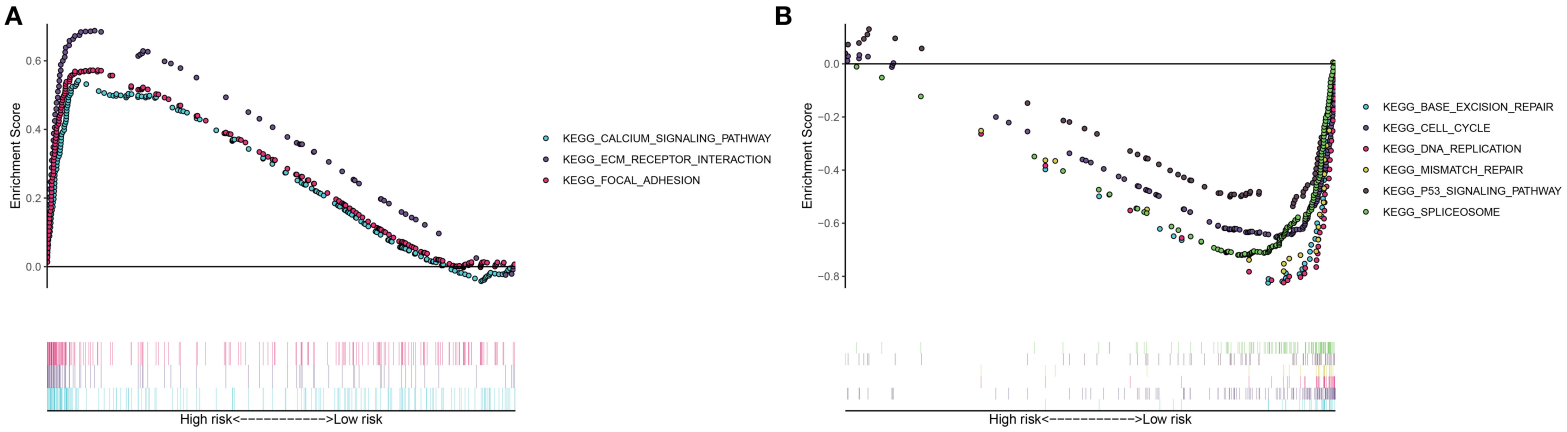
Gene set enrichment analysis (GSEA) for signaling pathways involved in high- and low-risk groups. **(A)** Highly enriched pathways in high-risk gastric cancer samples. **(B)** Highly enriched pathways in low-risk specimens.

This study evaluated the signaling pathways enriched by high- and low-risk samples via the GSEA in depth. Data indicated that calcium signaling pathway, ECM receptor interaction, and focal adhesion were highly enriched in high-risk samples (Figure 5A). In Figure 5B, base excision repair, cell cycle, DNA replication, mismatch repair, P53 signaling pathway, as well as spliceosome were highly enriched in low-risk specimens.

### Association Between This Signature and Tumor Microenvironment

**Figure 6:**
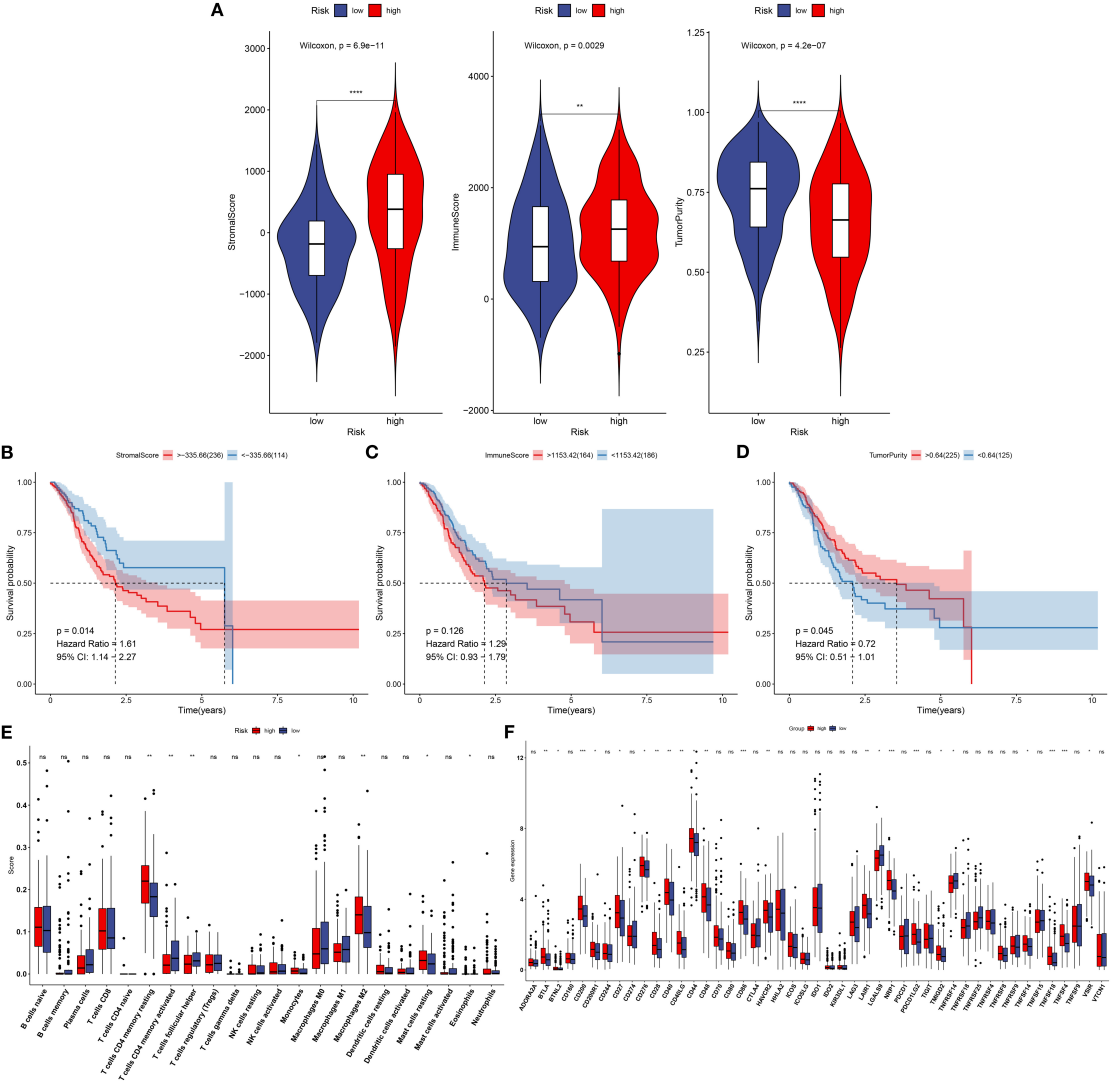
Association between this signature and tumor microenvironment in the training set. **(A)** Violin plots for stromal score, immune score, and tumor purity of high- and low-risk specimens via the ESTIMATE. **(B)** Kaplan–Meier curves of high- and low-stromal score groups. **(C)** Kaplan–Meier curves of high- and low-immune score groups. **(D)** Kaplan–Meier curves of high- and low-tumor purity groups. **(E)** Box plots for infiltration levels of immune cells in high- and low-risk samples through the CIBERSORT. **(F)** Box plots for expression of immune checkpoints in high- and low-risk samples. Ns, not significant. ^*^p < 0.05; ^**^p < 0.01; ^***^p < 0.001; ^****^p < 0.0001.

This study next probed the association between this signature and tumor microenvironment in the training set. In Figure 6A, high-risk specimens presented an increased stromal score (*p* = 6.9e−11) and immune score (*p* = 0.0029) than low-risk subjects. Meanwhile, high-risk subjects displayed distinctly lowered tumor purity in comparison with those with low risk (*p* = 4.2e−07). Prognostic values of stromal score, immune score, and tumor purity were then evaluated in gastric cancer. As a result, high stromal score was distinctly related to poorer prognosis than low stromal score (*p* = 0.014; Figure 6B). No significant difference in survival was found between high- and low-immune score groups (*p* = 0.126; Figure 6C). Moreover, high tumor purity was distinctly associated with prolonged survival duration (*p* = 0.045; Figure 6D). We further assessed whether the signature was in association with immune cell infiltrations in gastric cancer tissues from TCGA dataset. Data were indicative that subjects with high risk exhibited increased infiltration levels of T-cell CD4 memory resting (*p* < 0.01), monocytes (*p* < 0.05), macrophages M2 (*p* < 0.01), and mast cells resting (*p* < 0.05) in Figure 6E. Moreover, high-risk subjects had reduced infiltration levels of T-cell CD4 memory activated as well as T-cell follicular helper (both *p* < 0.01) than those with low risk. Furthermore, we found that high risk was characterized by increased expression of immune checkpoints including BTLA (*p* < 0.01), BTNL2 (*p* < 0.05), CD200 (*p* < 0.001), CD200R1 (*p* < 0.05), CD27 (*p* < 0.05), CD276 (*p* < 0.05), CD28 (*p* < 0.01), CD40 (*p* < 0.01), CD40LG (*p* < 0.01), CD44 CD40 (*p* < 0.05), CD48 (*p* < 0.01), CD40 (*p* < 0.01), CD86 (*p* < 0.001), HAVCR2 (*p* < 0.01), LAIR1 (*p* < 0.01), NRP1 (*p* < 0.001), PDCD1LG2 (*p* < 0.001), TMIGD2 (*p* < 0.05), TNFSF14 (*p* < 0.05), TNFSF18 (*p* < 0.001), TNFSF4 (*p* < 0.001), and VSIR (*p* < 0.05; Figure 6F).

### Correlation Between This Signature and Drug Sensitivity

**Figure 7:**
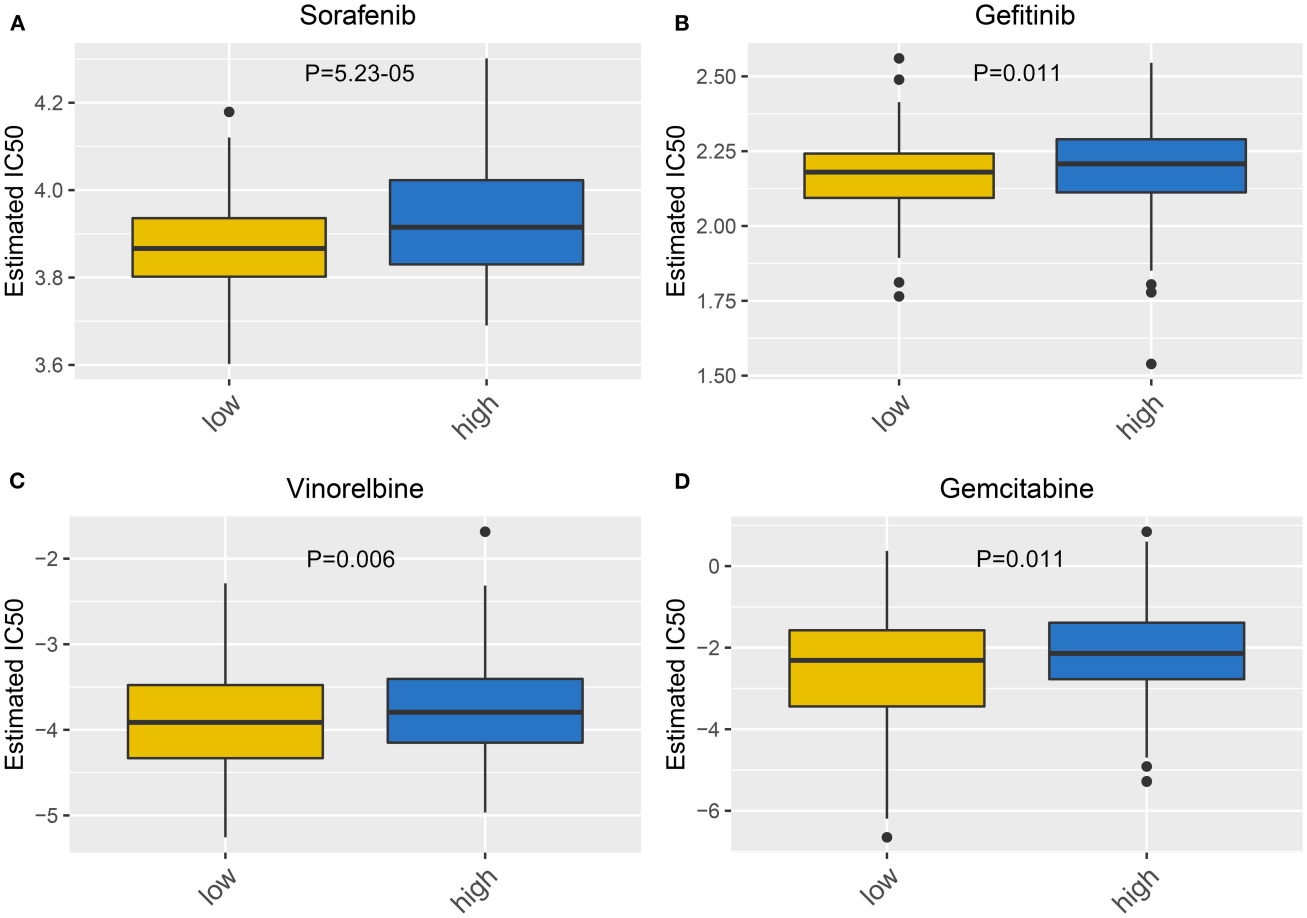
Correlation between this signature and drug sensitivity. Box plots for estimated IC_50_ of **(A)** sorafenib, **(B)** gefitinib, **(C)** vinorelbine, and **(D)** gemcitabine between high- and low-risk gastric cancer specimens.

We further evaluated the sensitivity to chemotherapy drugs between high- and low-risk groups. Our data were indicative of increased IC_50_ values of sorafenib (*p* = 5.23–05; Figure 7A), gefitinib (*p* = 0.011; Figure 7B), vinorelbine (*p* = 0.006; Figure 7C), and gemcitabine (*p* = 0.011; Figure 7D) in specimens with high risk than those with low risk. Hence, low-risk specimens were more sensitive to sorafenib, gefitinib, vinorelbine, and gemcitabine than high-risk specimens.

### Establishment of a Nomogram Integrating Age, Stage, and Risk Score

**Figure 8:**
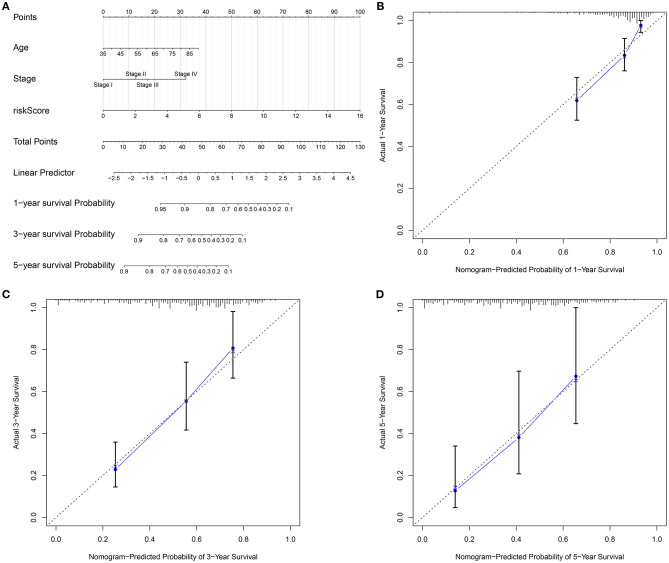
Establishment of a nomogram for gastric cancer in the training set. **(A)** A nomogram integrating age, stage, as well as signature in prediction of 1-, 3-, and 5-year outcomes in gastric cancer. The calibration curves for the relationships between **(B)** 1-, **(C)** 3-, and **(D)** 5-year survival predicted by this nomogram and actual clinical outcomes.

To personally predict the prognosis of each subject, a nomogram was established via integrating age, stage, and gene signature, which could be predictive of 1-, 3-, and 5-year survival probability (Figure 8A). Through confirmation of these calibration curves, 1-, 3-, and 5-year clinical outcomes by this nomogram exhibited high consistency with actual clinical outcomes for gastric cancer subjects in the training set (Figures 8B–D).

## Discussion

This study developed an RBP-based signature in the prediction of gastric cancer patients' survival. Subjects with high risk presented an unacceptable clinical outcome. Following verification, this signature was independently predictive of prognosis of patients. Moreover, it was distinctly related to immune microenvironment and sensitivity to chemotherapy drugs. Hence, this RBP-based signature may be a promising tool for predicting clinical outcomes and guiding clinical therapy in gastric cancer.

The molecular heterogeneity features between high- and low-risk patients were further analyzed. We found that calcium signaling pathway, ECM receptor interaction, and focal adhesion were highly activated in high-risk samples. Previously, calcium facilitates gastric carcinoma progress through calcium-sensing receptor as well as TRPV4 (27). Furthermore, VPAC1 and TRPV4 channels may accelerate gastric cancer progress by relying on calcium (28). The ECM receptor contributes to carcinogenesis, progress, and unfavorable survival in gastric cancer (29). Focal adhesion-related proteins are independently predictive of pessimistic clinical outcomes in gastric cancer (30). Meanwhile, activation of base excision repair, cell cycle, DNA replication, mismatch repair, P53 signaling pathway, as well as spliceosome was detected in low-risk specimens. The clinical implications of DNA repair like base excision repair and mismatch repair have been confirmed in gastric cancer (31). Deregulation of p53 pathway induces malignant biological properties for gastric cancer cells (32).

Immune cell ingredients contribute to gastric cancer initiation and progression. Moreover, immune escape exerts a critical role in tumorigenesis. Immune infiltration levels distinctly affect patients' survival. Tumor immune microenvironment that contains stromal and immune cells exhibits an association with immunotherapy response (5). Immune cells are correlated with tumor invasion and metastases. Stromal cells present close relationships with tumor growth, progression, response to chemotherapy, as well as recurrence. This study demonstrated that high-risk subjects had increased immune and stromal scores than those with low risk. Consistently, Mao et al. (33) found that subjects with high stromal scores presented unfavorable clinical outcomes. At present, novel immunotherapies like anti-PD-1 and anti-PD-L1 have been applied in gastric cancer. Nevertheless, only a minority of subjects benefit from immunotherapies. The compositions in the immune microenvironment are key determinants for prognoses and response to immunotherapies (34). Herein, this study comprehensively analyzed the correlations between immune cell infiltrations and this signature via the CIBERSORT algorithm. High-risk subjects presented increased infiltration levels of T-cell CD4 memory resting, monocytes, macrophage M2, and mast cells resting, and had reduced infiltration levels of T-cell CD4 memory activated as well as T-cell follicular helper than those with low risk. Moreover, we found that high risk was characterized by increased expression of immune checkpoints including BTLA that was expressed in B and T lymphocytes, BTNL2 that was expressed in antigen-processing and presentation cells, CD200 that was mainly expressed in B and T lymphocytes, CD200R1 that was expressed in myeloid lineage cells, CD27 that was expressed in T cells, CD276 that was expressed in cancer cells, CD28 that was expressed in T cells, CD40 that was expressed in antigen-presenting cells, CD40LG that was expressed in T cells, CD44 that was expressed in T cells, CD48 that was expressed in lymphocytes and dendritic cells, CD86 that was expressed in antigen-presenting cells, HAVCR2 that was expressed in T cells, LAIR1 that was expressed in natural killer cells, T cells, and B cells, NRP1 that was expressed in cancer cells, PDCD1LG2 that was expressed in T cells and dendritic cells, TMIGD2 that was expressed in T cells, TNFSF14 that was expressed in T cells, TNFSF18 that was expressed in T cells, TNFSF4 that was expressed in T cells, and VSIR that was expressed in T cells. These data were indicative of this signature being closely related to immunotherapy.

For advanced subjects, surgical resection followed by auxiliary chemotherapy is a major therapeutic strategy. In recent years, a few clinical trials of postoperative chemotherapy have been launched in gastric cancer (35–37). Miserably, response to chemotherapy is relatively low on account of tumor heterogeneity (38). Our data indicated that subjects with low risk were more sensitive to sorafenib, gefitinib, vinorelbine, and gemcitabine than those with high risk. This RBP-based signature seems to be considered as a classification tool for making individualized therapeutic decisions. Furthermore, a nomogram was then developed for individualized clinical outcome prediction. This model also showed good predictive performance for 1-, 3-, and 5-year survival.

A few disadvantages of this study need to be pointed out. First, this was a retrospective study according to public datasets. In our future studies, we will present prospective multicenter clinical trials for validation of this RBP signature in predicting gastric cancer patients' survival. Second, activated signal pathways in high- and low-risk subgroups should be verified in further basic experiments. In future research, the molecular mechanisms of RBPs will be observed in gastric cancer. Furthermore, we will further validate the relationships of RBPs with immune microenvironment of gastric cancer, which could be used for guiding immunotherapy in clinical practice.

## Conclusion

This study developed and externally verified an independent RBP-based signature in the prediction of gastric cancer patients' survival. This signature was closely related to tumor microenvironment and chemosensitivity, assisting in the expanding of the applications of immunotherapy and chemotherapy. A nomogram integrating this signature, age, and stage could offer individualized prediction of prognosis. Thus, this RBP signature may represent a prognostic stratification tool for gastric cancer.

## Data Availability Statement

The datasets presented in this study can be found in online repositories. The names of the repository/repositories and accession number(s) can be found in the article/Supplementary Material.

## Author Contributions

XL conceived and designed the study. ZQ conducted most of the experiments and data analysis, and wrote the manuscript. HJ and KJ participated in collecting the data and helped draft the manuscript. All authors reviewed and approved the manuscript.

## Conflict of Interest

The authors declare that the research was conducted in the absence of any commercial or financial relationships that could be construed as a potential conflict of interest.

## Supplementary Material

Supplementary Table 1 Clinical information of gastric cancer patients from TCGA database.

Supplementary Table 2 The clinical information of gastric cancer patients in the GSE84437 dataset.

Supplementary Table 3 A list of 1542 RBPs.

## Abbreviations

RBP: RNA-binding protein
TCGA: the cancer genome atlas
GEO: gene expression omnibus
LASSO: least absolute shrinkage and selection operator
ROC: receiver operating characteristic curve
AUC: area under the curve
HR: hazard ratio
CI: confidence interval
GSEA: gene set enrichment analysis
ESTIMATE: estimation of STromal and immune cells in MAlignant tumour tissues using expression data
CIBERSORT: Cell type identification by estimating relative subsets of RNA transcripts
GDSC: genomics of drug sensitivity in cancer
IC_50_: half maximal inhibitory concentration.
